# P2X7 receptor regulates leukocyte infiltrations in rat frontoparietal cortex following status epilepticus

**DOI:** 10.1186/1742-2094-7-65

**Published:** 2010-10-12

**Authors:** Ji-Eun Kim, Hea Jin Ryu, Seong-Il Yeo, Tae-Cheon Kang

**Affiliations:** 1 Department of Anatomy and Neurobiology, Institute of Epilepsy Research, College of Medicine, Hallym University, Chunchon 200-702, South Korea

## Abstract

**Background:**

In the present study, we investigated the roles of P2X7 receptor in recruitment and infiltration of neutrophil during epileptogenesis in rat epilepsy models.

**Methods:**

Status epilepticus (SE) was induced by pilocarpine in rats that were intracerebroventricularly infused with either saline, 2',3'-O-(4-benzoylbenzoyl)-adenosine 5'-triphosphate (BzATP), adenosine 5'-triphosphate-2',3'-dialdehyde (OxATP), or IL-1Ra (interleukin 1 receptor antagonist) prior to SE induction. Thereafter, we performed immunohistochemical studies for myeloperoxidase (MPO), CD68, interleukin-1β (IL-1β), monocyte chemotactic protein-1 (MCP-1) and macrophage inflammatory protein-2 (MIP-2).

**Results:**

In saline-infused animals, neutrophils and monocytes were observed in frontoparietal cortex (FPC) at 1 day and 2 days after SE, respectively. In BzATP-infused animals, infiltrations of neutrophils and monocytes into the FPC were detected at 12 hr and 1 day after SE, respectively. In OxATP-infused animals, neutrophils and monocytes infiltrated into the FPC at 1 day and 2 days after SE, respectively. However, the numbers of both classes of leukocytes were significantly lower than those observed in the saline-infused group. In piriform cortex (PC), massive leukocyte infiltration was detected in layers III/IV of saline-infused animals at 1-4 days after induction of SE. BzATP or OxATP infusion did not affect neutrophil infiltration in the PC. In addition, P2X7 receptor-mediated MCP-1 (released from microglia)/MIP-2 (released from astrocytes) regulation was related to SE-induced leukocyte infiltration in an IL-1β-independent manner.

**Conclusions:**

Our findings suggest that selective regulation of P2X7 receptor-mediated neutrophil infiltration may provide new therapeutic approaches to SE or epilepsy.

## Background

Epilepsy is a chronic condition characterized by the presence of spontaneous episodes of abnormal excessive neuronal discharges that result in specific patterns of neuron loss in various brain regions, particularly in the hippocampus [1,2]. Recent reports have emphasized that chronic epilepsy is a prolonged inflammatory condition, and that epileptic activity rapidly increases synthesis and release of various cytokines in rodent brain involved in seizure onset and generalization [3-7]. Release of cytokines affects turnover and release of various neurotransmitters and expression of neuropeptides and neurotrophic factors, and alters synaptic transmission and ionic currents in several rodent forebrain regions; and therefore appears to be directly involved in neuronal network excitability [6,7]. On the other hand, cytokines act on endothelial cells to change the permeability of the blood-brain barrier (BBB), with resulting significant effects on neuronal viability and excitability [8-11].

Pilocarpine (PILO) acts on muscarinic receptors. Both M1 and M2 receptors appear to be involved, however M1 receptors mediate most proepileptogenic actions [12]. The PILO-induced status epilepticus (SE) model replicates the cell type-specific pattern of neuron loss and axon reorganization found in many patients with temporal lobe epilepsy [13,14]. It also replicates a common clinical history of patients with temporal lobe epilepsy [15], in that a brain injury precedes a seizure-free latent period before spontaneous, recurrent seizures begin. Furthermore, PILO-induced SE affects WBC infiltration, cytokine levels, and BBB integrity [16]. Therefore, this model is useful for study not only epileptogenesis, but also inflammatory responses induced by SE.

The immune system in the brain is in part isolated from the systemic immune system by the BBB, and microglia are generally the only inflammatory cells within the brain. However, recent reports suggest that blood-derived inflammatory cells, including neutrophils and monocytes, infiltrate the brain under certain pathological conditions [17-20]. Infiltrating leukocytes accelerate local inflammatory processes through generation of toxic free radicals, release of proteolytic enzymes, and generation of proinflammatory cytokines [21-24]. Chemokines contribute to recruitment of leukocytes [25-27]. Chemokines such as monocyte chemotactic protein-1 (MCP-1) and macrophage inflammatory protein-2 (MIP-2) are undetectable or present at low levels under physiological conditions, and show transient increases under pathological conditions. Neurons, microglia and astrocytes produce MCP-1 or MIP-2 when incubated with pro-inflammatory cytokines, such as tumor necrosis factor-α (TNF-α) and/or interleukin-1β (IL-1β) or after injury [28-30]. The P2X7 receptor, an ATP-ligand channel, has attracted much attention as a modulator of inflammatory pathways in the brain, since the P2X7 receptor is upregulated after acute brain injury and in chronic neurological diseases [31-34], and releases cytokines/chemokines from neuroglia [35-37]. With respect to these P2X7 receptor functions, P2X7-mediated chemokine release is likely involved in neutrophil infiltration, although the mechanisms of neutrophil infiltration into brain parenchyma are still unknown. Therefore, we investigated the roles of the P2X7 receptor in recruitment and infiltration of neutrophil during epileptogenesis in rat epilepsy models provoked by PILO-induced SE.

## Methods

### Experimental animals

This study utilized the progeny of Sprague-Dawley (SD) rats (male, 9 - 11 weeks old) obtained from the Experimental Animal Center, Hallym University, Chunchon, South Korea. The animals were provided with a commercial diet and water *ad libitum *under controlled temperature, humidity and lighting conditions (22 ± 2°C, 55 ± 5% and a 12:12 light/dark cycle with lights). Procedures involving animals and their care were conducted in accord with our institutional guidelines that comply with NIH Guide for the Care and Use of Laboratory Animals (NIH Publications No. 80-23, 1996). In addition, we have made all efforts to minimize the number of animals used and their suffering.

### ICV drug infusion

Rats were divided into four groups treated with: (1) vehicle (saline), (2) 2',3'-O-(4-benzoylbenzoyl)-adenosine 5'-triphosphate (BzATP, P2X7 receptor agonist, 5 mM, Sigma), (3) adenosine 5'-triphosphate-2',3'-dialdehyde (OxATP, P2X7 receptor antagonist, 5 mM, Sigma) and (4) interleukin 1 receptor antagonist (IL-1Ra, 5 μg/ml, R&D systems). The dosage of each compound or IL-1Ra was determined as the highest dose that induced SE of comparable severity in 100% of animals with 5% mortality in a preliminary study. Animals were anesthetized (Zolretil, 50 mg/kg, I.M. Virbac Laboratories, France) and placed in stereotaxic frames. For osmotic pump implantation, holes were drilled through the skull to introduce a brain infusion kit 1 (Alzet, USA) into the right lateral ventricle (1 mm posterior; 1.5 mm lateral; - 3.5 mm depth; flat skull position with bregma as reference), according to the atlas of Paxinos and Watson [38]. The infusion kit was sealed with dental cement and connected to an osmotic pump (1007D, Alzet, USA). The pump was placed in a subcutaneous pocket in the dorsal region. Animals received 0.5 μl/hr of vehicle or compound for 1 week [39-41]. Therefore, the doses of BzATP, OxATP and IL-1Ra were 43 μg, 30 μg and 0.06 μg/day per animal, respectively. The compounds were infused beginning immediately after surgery. Since the number of neutrophils in brain parenchyma peaked at 2-3 days after SE in our preliminary study, we chose this time point. Thus, our experimental schedules were designed to inhibit the function of P2X7 receptor and IL-1β from at least 3 days prior to SE to at least 4 days after SE, when neutrophil infiltration peaked.

### Seizure induction

Three days after surgery, rats were treated with PILO (380 mg/kg, i.p.) 20 min after methylscopolamine (5 mg/kg, i.p.). Using this treatment paradigm, behavioral seizures typically began within 20-40 min. Approximately 80% of PILO-treated rats showed acute behavioral features of SE (including akinesia, facial automatisms, limbic seizures consisting of forelimb clonus with rearing, salivation, masticatory jaw movements, and falling). We used a 2-hr SE rat model, because > 90% of rats that we monitored in previous studies [42-44] displayed spontaneous, recurrent seizures within 1-3 months after PILO-induced SE. Diazepam (10 mg/kg, i.p.) was administered 2 hours after onset of SE and repeated, as needed. The rats were then observed 3 - 4 hours a day in a vivarium for general behavior and occurrence of spontaneous seizures. At designated time points (12 hrs, 1, 2, 3 and 4 days after SE; n = 30, respectively), animals were killed and used for immunohistochemistry. Rats not expriencing SE (those which showed only acute seizure behaviors during 10 - 30 min, n = 22) and age-matched normal rats were used as controls (n = 15).

### Tissue processing

Animals were perfused transcardially with phosphate-buffered saline (PBS) followed by 4% paraformaldehyde in 0.1 M phosphate buffer (PB, pH 7.4) under urethane anesthesia (1.5 g/kg, i.p.). The brains were removed, and postfixed in the same fixative for 4 hrs. The brain tissues were cryoprotected by infiltration with 30% sucrose overnight. Thereafter, the entire hippocampus was frozen and sectioned with a cryostat at 30 μm and consecutive sections were placed in six-well plates containing PBS. For stereological study, every sixth section in a series through the entire hippocampus was used in some animals.

### Immunohistochemistry

The sections were first incubated with 3% bovine serum albumin in PBS for 30 min at room temperature. Sections were then incubated in rabbit anti-myeloperoxidase (MPO) IgG (diluted 1:100, Thermo fisher scientific, USA), mouse anti-CD68 IgG (diluted 1:100, Abcam, USA), goat anti-IL-1β IgG (diluted 1:100, R&D system), rabbit anti-MCP-1 IgG (diluted 1:100, Abcam, USA) or rabbit anti-MIP-2 IgG (diluted 1:100, Invitrogen, USA) in PBS containing 0.3% Triton X-100 overnight at room temperature. The sections were washed three times for 10 min with PBS, incubated sequentially, in biotinylated goat anti-rabbit IgG, anti-mouse IgG or rabbit anti-goat IgG (Vector, Burlingame, CA, USA) and in an avidin-biotin-complex (ABC, Vector Laboratories, Burlingame, CA, USA), diluted 1:200 in the same solution as the primary antiserum. Between incubations, the tissues were washed with PBS three times for 10 min each. The sections were visualized with 3,3'-diaminobenzidine (DAB) in 0.1 M Tris buffer and mounted on gelatin-coated slides. The immunoreactions were observed under the Axiophot microscope (Carl Zeiss, Germany). All images were captured using an Axiocam HRc camera and Axio Vision 3.1 software.

### Multiple immunofluorescence staining

To identify the morphological changes induced by SE in the same hippocampal tissue, double immunofluorescent staining was performed. Brain tissues were incubated in mixture of rabbit anti-Iba-1 IgG (diluted 1:100, Biocare medical, USA)/goat anti-IL-1β IgG (diluted 1:100), rabbit anti-MCP-1 IgG (diluted 1:100)/rabbit anti-Iba-1 IgG (diluted 1:100), rabbit anti-GFAP (diluted 1:200, Chemicon, USA)/goat anti-MIP-2 IgG (diluted 1:500) or rabbit anti-CCR2 IgG (dilution 1:100, Abcam, USA)/goat anti-MIP-2 IgG (diluted 1:500) overnight at room temperature. After washing three times for 10 minutes with PBS, sections were also incubated in a mixture of FITC- and Cy3-conjugated secondary antisera (1:200, Amersham, USA) or streptavidin (1:200, Vector, USA) for 1 hr at room temperature. For detection of rabbit anti-MCP-1 and rabbit anti-Iba-1, we applied tyramide amplification methods [45]. The sections were washed three times for 10 min with PBS, and mounted on gelatin-coated slides. For nuclei counterstaining, we used Vectashield mounting medium with DAPI (Vector, USA). All images were captured using an Axiocam HRc camera and Axio Vision 3.1 software (Carl Zeiss, Munchen-Hallbergmoos, Germany). Figures were compiled using Adobe PhotoShop 7.0 (San Jose, CA). Manipulation of the images was restricted to threshold and brightness adjustments to the entire image.

### Quantification of data and statistical analysis

For quantification of immunohistochemical data, areas were selected from brain tissues approximately 0.2 - 3.8 mm from bregma based on the rat brain in stereotaxic coordinates [38]. Cells in 2 - 4 regions (1 × 10^5 ^μm^2^) from each section were counted on 20× images. Results are presented as means ± SD of 15 - 25 regions from five animals. All immunoreactive cells were counted regardless the intensity of labeling. Cell counts were performed by two different investigators who were blind to the classification of tissues. All data obtained from the quantitative measurements were analyzed using one-way ANOVA to determine statistical significance. Bonferroni's test was used for post-hoc comparisons. A p-value below 0.05 or 0.01 was considered statistically significant [42,43].

## Results

Restricted blood-derived leukocyte infiltration was observed in the frontoparietal cortex (FPC) and piriform cortex (PC) during the time-window applied in the present study. Therefore, we describe the infiltration patterns of blood-derived leukocyte in both cortical regions.

### Neuronal damage

**Figure 1:**
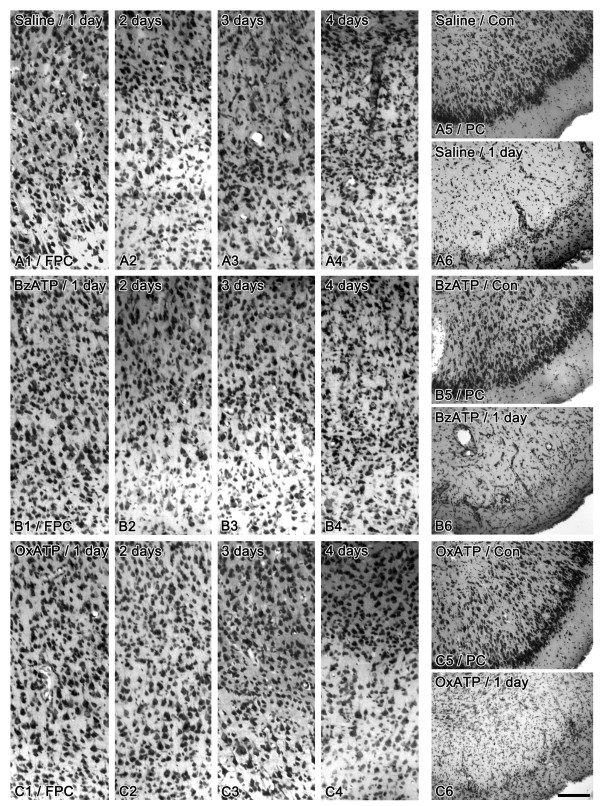
**Neuronal damage following SE**. (A) Saline-infused animal, (B) BzATP-infused animal, (C) OxATP-infused animal. In saline-infused animals, no apparent neuronal loss is observed in the FPC at 1 day after SE (A1). Two days after SE, neuronal loss is detected in layers IV-V (A2). Three-to-four days after SE, widespread neuronal damage is detected in the layers II-V of the FPC (A3-4). In BzATP-treated animals, neuronal loss is detected at 1 day after SE (B1-4). In OxATP-treated animals, neuronal loss is detected at 4 days after SE (C1-4). In PC, the severe neuronal loss accompanied by edematous findings is detected in layers II-IV at 1 day after SE in every group (A5-6, B5-6 and C5-6). Bar = 100 μm.

In saline-infused animals, no apparent neuronal loss was observed in the FPC at 1 day after SE (Figure 1A1). Two days after SE, neuronal loss was detected in layers IV-V (Figure 1A2). Three-four days after SE, widespread neuronal damage was detected in layers II-V of the FPC (Figure 1A3-4). In BzATP-treated animals, neuronal loss was detected at 1 day after SE (Figures 1B1-4). In OxATP-treated animals, neuronal loss was detected at 4 days after SE (Figures 1C1-4). In contrast to FPC, neuronal damage in the PC was similarly observed in every group. Briefly, severe neuronal loss accompanied by edematous findings was detected in layers II-IV of the PC at 1 day after SE (Figures 1A5-6, 1B5-6 and 1C5-6).

### Neutrophil infiltration after SE

**Figure 2:**
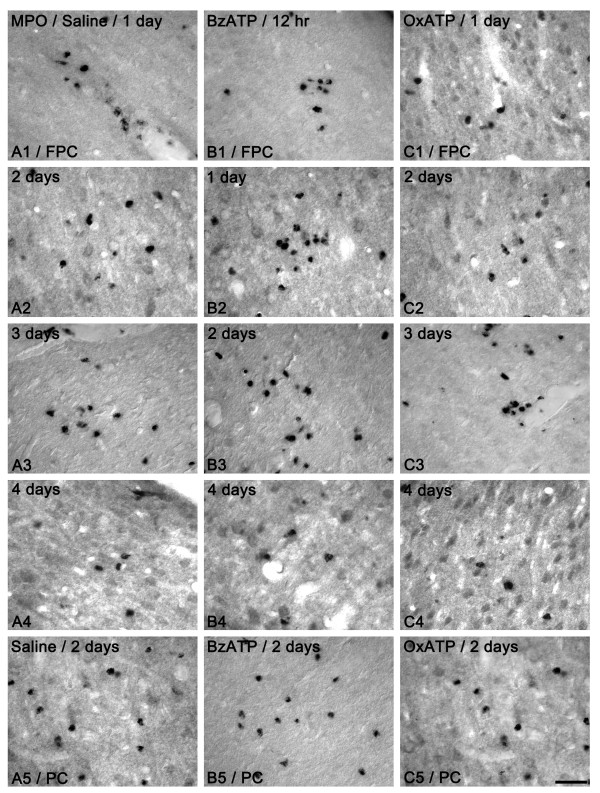
**MPO-positive neutrophil infiltration following SE**. (A) Saline-infused animal, (B) BzATP-infused animal, (C) OxATP-infused animal. In saline-infused animals, neutrophils are observed in the perivascular parenchyma of the FPC at 1 day after SE (A1). Two-to- three days after SE, the number of neutrophils is increased in this region (A2-3). Four days after SE, the number of neutrophils is markedly reduced (A4). In BzATP-infused animals, infiltration of neutrophils into the FPC is detected at 12 hr after SE (B1). At 1-3 days after SE, the numbers of neutrophils is increased (B2-3). Four days after SE, the number of neutrophils is markedly reduced (B4). In OxATP-infused animals, neutrophils infiltrate into the FPC at 1 day after SE (C1). Two-to-three days after SE, the number of neutrophils is increased in the FPC (C2-3). Four days after SE, the number of neutrophils is markedly reduced (C4). In PC, massive neutrophil infiltration is detected in the layer III/IV of the saline-infused animals at 1-2 days after SE (A5). BzATP or OxATP infusion did not affect neutrophil infiltration in the PC (A5, B5 and C5). Bar = 50 μm.

**Figure 3:**
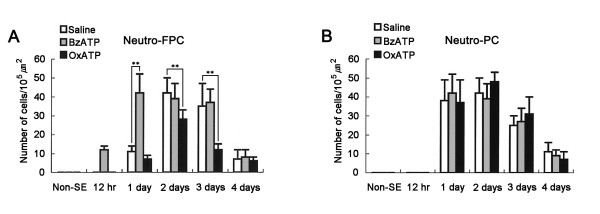
**Quantitative analysis of neutrophil infiltration in FPC (A) and PC (B) following SE**. Significant differences from saline-infused animals, **P < 0.01.

In saline-infused animals, MPO-positive neutrophils were observed in the perivascular parenchyma of the FPC at 1 day after SE (Figure 2A1). Two-three days after SE, the number of MPO-positive neutrophils had increased in this region (Figures 2A2-3 and 3A). Four days after SE, the number of MPO-positive neutrophils was markedly reduced (Figure 2A4 and 3A). In BzATP-infused animals, infiltration of MPO-positive neutrophils into the FPC was detected at 12 hr after SE (Figures 2B1). At 1-3 days after SE, the number of MPO-positive neutrophils had increased (Figures 2B2-3 and 3A). Four days after SE, the number of MPO-positive neutrophils was markedly reduced (Figures 2B4 and 3A). In OxATP-infused animals, MPO-positive neutrophils showed infiltration into the FPC at 1 day after SE (Figure 2C1). Two-to-three days after SE, the number of MPO-positive neutrophils was increased in the FPC (Figure 2C2-3). Four days after SE, the number of MPO-positive neutrophils was markedly reduced (Figures 2C4 and 3A). Although the temporal patterns of neutrophil infiltration were similar, the number of MPO-positive neutrophils was significantly lower than that observed in saline-infused group (Figure 3A). In the PC, massive neutrophil infiltration was detected in layer III/IV of saline-infused animals at 1-2 days after SE (Figures 2A5 and 3B). BzATP or OxATP infusion did not affect neutrophil infiltration in the PC (Figures 2B5, 2C5 and 3B). These findings indicate that P2X7 receptor activation may play an important role in neutrophil infiltration into the FPC after SE.

### Monocyte infiltration after SE

**Figure 4:**
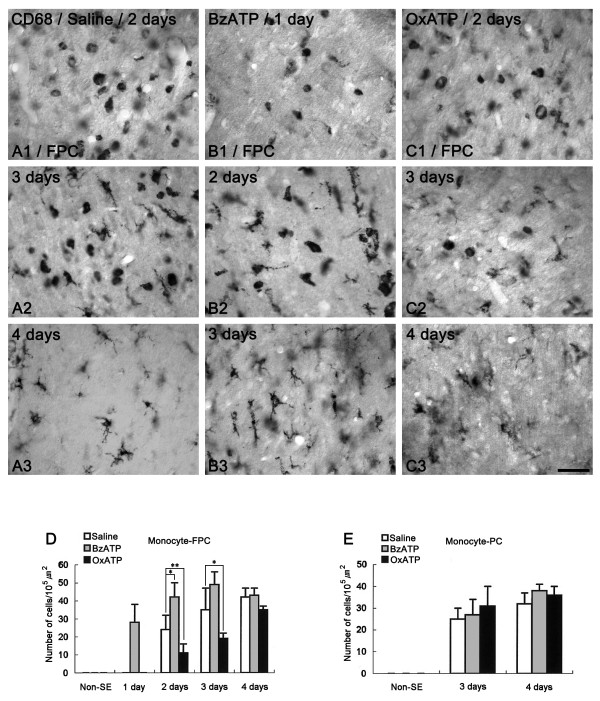
**CD 68-positive monocyte infiltration following SE**. (A) Saline-infused animal, (B) BzATP-infused animal, (C) OxATP-infused animal. In saline-infused animals, a few round-shaped monocytes are observed near blood vessels in the FPC at 2 days after SE (A1). Three-to-four days after SE, the number of CD68-positive cells is significantly increased in the FPC, and the shape is changed to a ramified form (A2-3). In BzATP-infused animals, monocytes are observed in the FPC at 1 day after SE (B1). Two-to-three days after SE, the number of CD68-positive cells is significantly increased, and their morphologies are changed to a ramified form (B2-3). In OxATP-infused animals, monocytes are observed in the FPC at 2 days after SE (C1). Three-to-four days after SE, the shape of the CD68-positive cells is changed to ramified forms, while the number of CD68-positive monocytes in this group is smaller than that in saline-infused animals (C2-3). Bar = 25 μm. (D-E) Quantitative analysis of monocyte infiltration in FPC (D) and PC (E) following SE. Significant differences from saline-infused animal, *P < 0.05 and **P < 0.01.

In saline-infused animals, a few round-shaped CD68-positive monocytes were observed near blood vessels in the FPC at 2 days after SE (Figure 4A1). Three-to-four days after SE, the number of CD68 positive cells had significantly increased in the FPC (Figures 4A2-3 and 4D). Furthermore, the shape of the CD68-positive cells had changed to a ramified form (Figures 4A2-3). In BzATP-infused animals, CD68-positive monocytes were observed in the FPC at 1 day after SE (Figure 4B1). Two-to-three days after SE, the number of CD68-positive cells had significantly increased, and their morphology had changed to a ramified form (Figures 4B2-3 and 4D). In OxATP-infused animals, CD68-positive monocytes were observed in the FPC at 2 days after SE (Figure 4C1). Three-to-four days after SE, the shape of CD68-positive cells had changed to a ramified form, while the number of CD68-positive monocytes in this group was smaller than that in saline-infused animals (Figures 4A2-3 and 4D). In the PC, CD68-positive cell infiltration was detected in layer III/IV of saline-infused animals at 3 days after SE (Figures 4E). The morphology of the CD68-positive cells had changed from a round shape to a ramified form at 4 days after SE (data not shown). BzATP or OxATP infusion did not affect CD68-positive monocyte infiltration in the PC (Figures 4E). These findings indicate that P2X7 receptor activation may also play a role in monocyte infiltration into the FPC after SE.

### The effect of IL-1Ra on leukocyte infiltration into the FPC after SE

**Figure 5:**
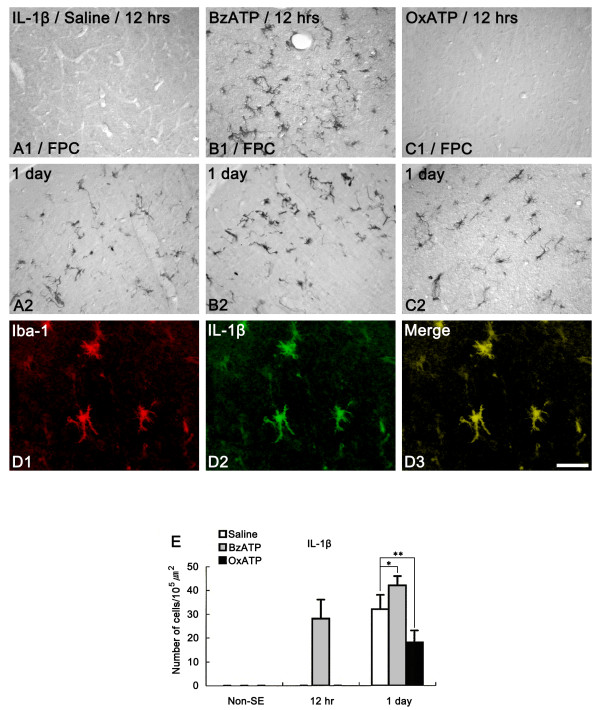
**IL-1β expression following SE**. (A) Saline-infused animal, (B) BzATP-infused animal, (C) OxATP-infused animal. In saline-infused animals, IL-1β-immunoreactive cells are detected in the FPC at 1 day after SE (A1-2). In BzATP-infused animals, IL-1β-immunoreactive microglia are observed at 12 hr after SE (B1-2). In OxATP-infused animals, IL-1β-immunoreactive microglia are observed at 1 days after SE (C1-2). Double immunofluorescent study shows that IL-1β-immunoreactive cells are Iba-1-positive microglia (D1-3). Bar = 50 (panels A, B and C) and 25 (panels D) μm. (D) Quantitative analysis of IL-1β-immunoreactive cells in FPC following SE. Significant differences from saline-infused animals, *P < 0.05 and **P < 0.01.

**Figure 6:**
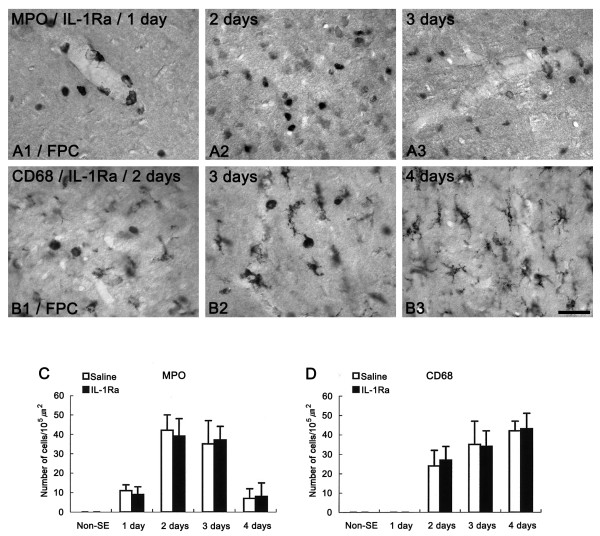
**The effect of IL-1Ra infusion on leukocyte infiltration**. IL-1Ra infusion does not affect MPO-positive neutrophil (A1-3) and CD68-positive cell (B1-3) infiltration after SE. Bar = 25 μm. (C-D) Quantitative analyses of neutrophil (C) and monocyte (D) infiltrations in FPC following SE. There were no significant differences from saline-infused animals.

In the present study, saline-infused animals showed IL-1β-immunoreactive cell in the FPC at 1 day after SE (Figures 5A1-2). Double immunofluorescent study revealed that IL-1β-immunoreactive cells were Iba-1-positive microglia (Figures 5D1-3). In the BzATP-infused group, IL-1β-immunoreactive microglia were observed at 12 hr after SE (Figures 5B1-2). Furthermore, the number of IL-1β-immunoreactive microglia was higher than that observed in saline-infused animals at 1 day after SE (Figures 5E). In OxATP-infused animals, IL-1β-immunoreactive microglia were observed at 1 day after SE (Figures 5C1-2). However, the number of IL-1β-immunoreactive microglia was lower than that observed in saline-infused animals (Figures 5E). These findings simply indicate that P2X7 receptor antagonist may inhibit leukocyte infiltration via an IL-1β-mediated pathway. Therefore, in order to confirm a direct effect of the IL-1β system on neutrophil infiltration, we applied IL-1 receptor antagonist (IL-1Ra) prior to SE induction. Unexpectedly, IL-1Ra infusion did not affect neutrophil infiltration after SE (Figures 6A1-3 and 6C). Furthermore, IL-1Ra infusion did not attenuate SE-induced neuronal damages in the FPC, compared to saline-infused animals (data not shown). In IL-1Ra-infused animals, similar to saline-infused animals, MPO-positive neutrophils were observed in the perivascular parenchyma of the FPC at 1 day after SE. One-to-three days after SE, the number of MPO-positive neutrophils had increased in this region. Similar to neutrophil infiltration, IL-1Ra infusion did not affect CD68-positive cell infiltration. Briefly, a few round-shaped CD68 positive monocytes were observed near blood vessels in the FPC at 2 days after SE (Figure 6B1). Three-to-four days after SE, the number of CD68-positive cells had significantly increased in the FPC (Figures 6B2-3 and 6D). Furthermore, the shape of these CD68-positive cells had changed to a ramified form (Figures 6B2-3). These findings indicate that activation of the P2X7 receptor accelerates leukocyte infiltration into brain parenchyma in an IL-1β-independent manner.

### MCP-1 and MIP-2 expression after SE

**Figure 7:**
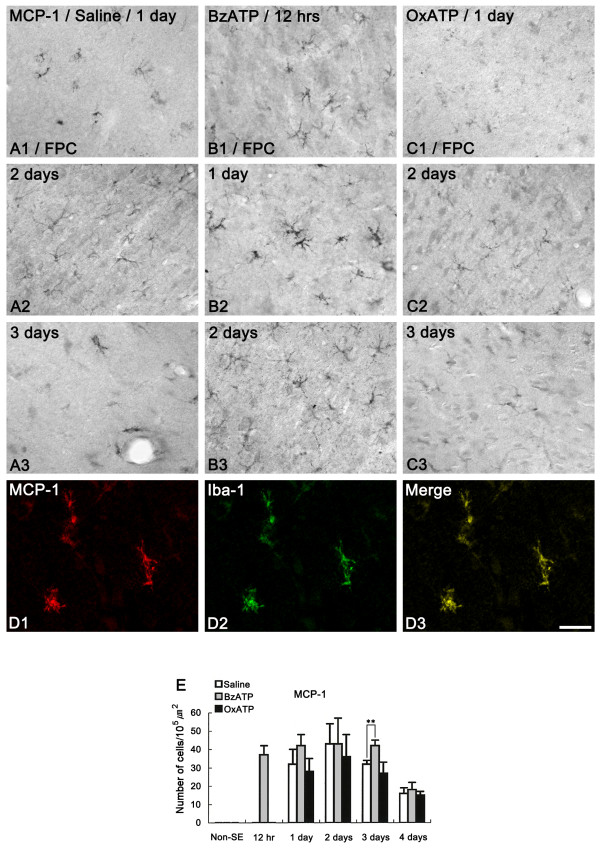
**MCP-1 expression following SE**. (A) Saline-infused animal, (B) BzATP-infused animal, (C) OxATP-infused animal. In saline-infused animals, MCP-1 immunoreactive cells are detected in the FPC at 1 day after SE (A1). The number of MCP-1-immunoreactive microglia is increased at 2 days after SE compared to that observed 1 day after SE (A2). The number of MCP-1-immunoreactive microglia is reduced at 3 days after SE (A3). In BzATP-infused animals, MCP-1 immunoreactivity is observed at 12 hr after SE (B1). The number of MCP-1 immunoreactive microglia is increased at 1-2 days after SE (B2-3). In OxATP-infused animals, changes in MCP-1 expression are similar to those in saline-infused animals (C1-3). Double immunofluorescent study shows that MCP-1 immunoreactive cells are Iba-1-positive microglia (D1-3). Bars = 50 μm (panels A, B and C) and 25 μm (panel D). (E) Quantitative analysis of MCP-1-immunoreactive cells in FPC following SE. Significant differences from saline-infused animals, **P < 0.01.

In saline-infused animals, MCP-1 immunoreactive cells were detected in the FPC at 1 day after SE (Figure 7A1). Double immunofluorescent studies revealed that MCP-1-immunoreactive cells were Iba-1-positive microglia (Figures 7D1-3). The number of MCP-1-immunoreactive microglia increased at 2 days after SE, compared to that observed at 1 day after SE (Figures 7A2 and 7E). Thereafter, the number of MCP-1-immunoreactive microglia showed a reduction at 3 days after SE (Figures 7A3 and 7E). In BzATP-infused animals, MCP-1 immunoreactivity was observed at 12 hr after SE (Figure 7B1). Furthermore, the number of MCP-1-immunoreactive microglia had increased at 1-2 days after SE (Figures 7B2-3 and 7E). In OxATP-infused animals, changes in MCP-1 expression were similar to those in saline-infused animals (Figures 7C1-3), while the number of MCP-1-immunoreactive microglia in this group was smaller (but not significantly) than that in saline-infused animals (Figure 7E). IL-1Ra infusion did not affect MCP-1 immunoreactivity after SE (data not shown).

**Figure 8:**
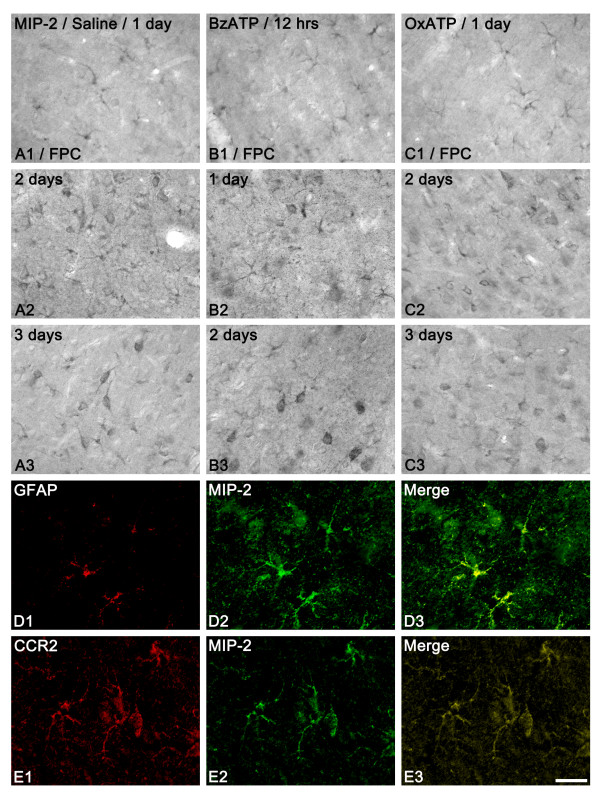
**MIP-2 expression following SE**. (A) Saline-infused animal, (B) BzATP-infused animal, (C) OxATP-infused animal. In saline-infused animals, astrocytes show MIP-2 immunoreactivity at 1 day after SE (A1 and D1-3). At 2-3 days after SE, both astrocytes and neurons show MIP-2 immunoreactivity (A2-3). In BzATP-infused animals, MIP-2 immunoreactivity is observed in astrocytes at 12 hr after SE (B1). At 1-2 days after SE, both astrocytes and neurons show MIP-2 immunoreactivity (B2-3). In OxATP-infused animals, MIP-2 immunoreactivity is observed in astrocytes at 1 day after SE (C1). At 2-3 days after SE, both astrocytes and neurons show MIP-2 immunoreactivity (C2-3). Double immunofluorescent study shows that astrocytes contain both MIP-2 (D1-3) and CCR2 (E1-3) immunoreactivities. Bars = 50 μm (panels A, B and C) and 25 μm (panels D and E) μm.

In saline-infused animals, astrocytes showed MIP-2 immunoreactivity at 1 day after SE (Figures 8A1 and 8D1-3). At 2-3 days after SE, both astrocytes and neurons showed MIP-2 immunoreactivity, which were colocalized with CCR2 (receptor for MCP-1) immunoreactivity (Figures 8A2-3 and 8E1-3). In BzATP infused animals, MIP-2 immunoreactivity was observed in astrocytes at 12 hr after SE (Figure 8B1). At 1-2 days after SE, both astrocytes and neurons showed MIP-2 immunoreactivity (Figures 8B2-3). In OxATP infused animals, MIP-2 immunoreactivity was observed in astrocytes at 1 day after SE (Figure 8C1). At 2-3 days after SE, both astrocytes and neurons showed MIP-2 immunoreactivity, while the number of MIP-2 immunoreactive cells in this group was smaller than that in saline-infused animals (Figures 8C2-3 and 9). IL-1Ra infusion did not affect MIP-2 immunoreactivity after SE (data not shown). These findings indicate that P2X7 receptor activation may accelerate up-regulation of MCP-1 and MIP-2 expression in the FPC, resulting in leukocyte infiltration.

**Figure 9:**
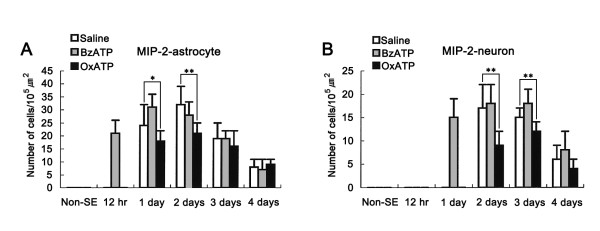
**Quantitative analyses of MIP-2 immunoreactivity in astroglial cells (A) and neurons (B) following SE**. Significant differences from saline-infused animal, *P < 0.05 and **P < 0.01.

## Discussion

SE rapidly increases synthesis and release of cytokines in various areas of rodent brain [3-7]. Furthermore, blood-derived leukocyte infiltration appears in brain parenchyma after SE. Neutrophil infiltration into brain parenchyma is transiently observed during the acute phase of SE (4 - 36 hr after SE) disappearing thereafter. Later, monocytes are found in brain parenchyma, and persist during epileptogenesis. However, B- and T-lymphocytes and NK cells are found strictly associated with brain microvessels and rarely in brain parenchyma after SE [46]. In the present study, apparent neuronal loss was observed in the FPC of saline-infused animals at 2-4 days after SE, when neutrophil infiltration was detected. BzATP infusion exacerbated neuronal death accompanied by acceleration of neutrophil infiltration, while OxATP infusion attenuated them. Leukocyte infiltration induces generation of reactive oxygen species (ROS), release of proteolytic enzymes, and synthesis of proinflammatory cytokines [21,47], which result in cell injury by peroxidation of polyunsaturated lipids, DNA damage, inhibition of glycolysis, oxidative phosphorylation by NADPH oxidase and myeloperoxidase, depletion of intracellular ATP and alterations of ATP-dependent ion pumps [48-52]. Therefore, our findings suggest that P2X7 receptor-mediated leukocyte infiltration (particularly neutrophil infiltration) may be a crucial factor in neuronal damage in the FPC following SE.

MCP-1 is primarily credited with recruitment of macrophage populations to sites of expression, but is also capable of acting as a T-cell and dendritic cell chemotactic stimulus [53,54]. In contrast, MIP-2 is required for efficient neutrophil or lymphocyte recruitment to brain parenchyma [55]. In the present study, MCP-1 immunoreactivity was detected in microglia, and CCR2 immunoreactivity was colocalized with MIP-2 immunoreactivity in astrocytes and neurons after SE. Furthermore, these SE-induced changes in chemokine expression were correlated with P2X7 receptor-mediated leukocyte infiltration and neuronal damage in the FPC. Indeed, recent studies have reported that MCP-1 recruits neutrophils into brain parenchyma via an unknown pathway [56,57] and that inhibition of P2X7 receptor reduces neutrophil infiltration [58]. Taken together, our findings indicate that P2X7 receptor may regulate MCP-1 expression/release in microglia, which may modulate MIP-2 expression/release in neurons and astrocytes via CCR2.

Since CD68 is a commonly used marker for peripheral monocytes and activated microglia [59-61], we cannot exclude the possibility that the CD68-positive cells with "ramified" morphologies are activated microglia. In the present study, however, spheroid CD68-positive cells are likely peripherally-derived monocytes in the early time windows. Therefore, it is obvious that P2X7 receptor activation may accelerate monocyte infiltration in the brain parenchyma following SE.

Recently, Peng et al. [58] reported that systemic administration of Brilliant blue G (BBG), a selective P2X7 receptor antagonist, resulted in improved motor recovery without evident toxicity. In addition, BBG directly reduced local activation of astrocytes and microglia, as well as neutrophil infiltration. They have suggested that attenuation of neutrophil invasion by BBG may be due to blockade of the P2X7 receptor in neutrophils themselves. Similar to this, the present study shows that OxATP infusion attenuates neuronal damage and leukocyte infiltration following SE. However, considering the inability of OxATP to cross the blood-brain barrier, it is unlikely that OxATP infusion could directly affect neutrophils in blood vessels. Therefore, P2X7 receptor-mediated chemokine release/expression may play a role in leukocyte infiltration rather than the direct effect of BzATP or OxATP on leukocytes.

IL-1β plays a role in development of neuronal cell death after traumatic, ischemic, excitotoxic, and seizure-induced brain injury [62-64]. IL-1β alone is capable of overriding the intrinsic resistance of the brain to leukocyte infiltration, resulting in acute cellular recruitment to brain parenchyma [65-69]. In the present study, BzATP infusion increased IL-1β expression induced by SE, compared to saline infusion. Since the P2X7 receptor modulates IL-1β release from glial cells [70-72], it is likely that inhibition of IL-1β by IL-1Ra infusion would reduce SE-induced neuronal death or neutrophil infiltration. Indeed, sustained IL-1β expression is able to drive localized, persistent leukocyte infiltration of brain parenchyma [56]. In the present study, unexpectedly, IL-1Ra infusion did not affect SE-induced leukocyte infiltration, even though IL-1β is a powerful regulator of chemokines in the rat brain [73]. Furthermore, compared to saline infusion, IL-1Ra infusion was not effective against SE-induced neuronal damage. These findings indicate that SE-induced leukocyte infiltration into brain parenchyma may be induced in an IL-1β-independent manner. However, it cannot be excluded that the dose of IL-1Ra used was insufficient to prevent SE-induced neuronal death and leukocyte infiltration. Because of the anti-convulsive effect of IL-1Ra [74], we applied the maximal dose of IL-1Ra that did not affect the PILO-induced seizure threshold in the present study. IL-1β inhibits astroglial glutamate re-uptake in an interleukin-1 receptor I- (IL-1RI) dependent manner [75-77]. Indeed, IL-1RI expression increases in neurons following SE [78]. Furthermore, IL-1β increases *N*-methyl-*D*-aspartate (NMDA) receptor activity through IL-1RI-mediated activation of Src kinase family-mediated tyrosine phosphorylation of NR2A/B, which results in increased intracellular Ca^2+ ^through an increase of its channel gating properties [74,79]. Therefore, IL-1β induces neuronal death in an NMDA receptor-dependent manner [7], promoting cross talk between proinflammatory and excitatory pathways [80]. Indeed, IL-1β expression is not capable of neurotoxicity by itself, but serves to lower the threshold for additional injury [81-84]. With respect to the previous studies described above, it is likely that below-anti-convulsive doses of IL-1Ra would be insufficient for prevention of SE-induced neuronal death and leukocyte infiltration. Therefore, the neuroprotective effect of IL-1Ra may be based on an anti-excitotoxic mechanism rather than anti-inflammatory pathways. Further studies are needed to elucidate the role of IL-1Ra in SE-induced neuronal damage and leukocyte infiltration.

In the present study, massive leukocyte infiltration was detected in the PC. However, BzATP, OxATP or IL-1Ra infusion did not affect leukocyte infiltration in this region. After SE, severe edema accompanied by neuronal and astroglial damage occurred in the PC [85]. In our preliminary study (Kim et al., in preparation for submission), because severe serum-protein extravasation was observed in layers III/IV and had spread to layer II at 3 days after SE, neuronal and astroglial damage in the PC was related to vasogenic edema. Therefore, it is likely that leukocyte infiltration in the PC may be related to vasogenic edema.

## Conclusions

Our findings suggest that inflammatory responses by leukocyte infiltration into the brain may be one of the crucial factors in SE-induced brain damage, and that P2X7 receptor-mediated MCP-1/MIP-2 regulation may play an important role in SE-induced leukocyte infiltration in an IL-1β-independent manner. Therefore, our findings suggest that selective regulation of P2X7 receptor functions may provide new therapeutic approaches to SE or epilepsy.

## Competing interests

The authors declare that they have no competing interests.

## Authors' contributions

JEK performed all experiments in the present study. JEK, HJR and SIY performed the immunohistochemistry and osmotic pump implantations. HJR and SIY helped in drafting the manuscript. JEK and TCK provided continuous intellectual input, evaluation and interpretation of data. TCK conceived, designed and coordinated the project, and drafted the manuscript. All authors read and approved the final manuscript.
